# Ecological lifestyle and gill slit height across sharks

**DOI:** 10.1098/rsos.231867

**Published:** 2024-05-29

**Authors:** Wade J. VanderWright, Jennifer S. Bigman, Anthony S. Iliou, Nicholas K. Dulvy

**Affiliations:** ^1^ Earth to Ocean Research Group, Department of Biological Sciences, Simon Fraser University, Burnaby, British Columbia V5A 1S6, Canada; ^2^ Alaska Fisheries Science Center, NOAA, Seattle, WA, USA

**Keywords:** Chondrichthyes, elasmobranch, fast–slow life history, metabolism

## Abstract

Metabolic morphology—the morphological features related to metabolic rate—offers broad comparative insights into the physiological performance and ecological function of species. However, some metabolic morphological traits, such as gill surface area, require costly and lethal sampling. Measurements of gill slit height from anatomically accurate drawings, such as those in field guides, offer the opportunity to understand physiological and ecological function without the need for lethal sampling. Here, we examine the relationship between gill slit height and each of the three traits that comprise ecological lifestyle: activity, maximum body size, and depth across nearly all sharks (*n* = 455). We find that gill slit heights are positively related to activity (measured by the aspect ratio of the caudal fin) and maximum size but negatively related to depth. Overall, gill slit height is best explained by the suite of ecological lifestyle traits rather than any single trait. These results suggest that more active, larger and shallower species (and endothermic species) have higher metabolic throughput as indexed by gill slit height (oxygen uptake) and ecological lifestyle (oxygen expenditure). We show that meaningful ecophysiological relationships can be revealed through measurable metabolic morphological traits from anatomically accurate drawings, which offers the opportunity to estimate class-wide traits for analyses of life history theory and the relationship between biodiversity and ecological function.

## 1. Introduction

Metabolic rate is thought to set the pace of life, from governing organismal developmental rates and life history traits to ecosystem processes such as biomass cycling [1]. This fundamental rate is typically measured indirectly by oxygen consumption over time in a controlled laboratory setting and is difficult to measure for large-bodied, free-swimming organisms such as sharks [2,3]. For sharks (and other fishes), the surface area of the gills is tightly correlated with metabolic rate such that individuals and species with large gill surface areas have higher oxygen consumption rates (i.e. metabolic rates) [4–6]. However, there are few estimates of metabolic rates for sharks and rays and gill surface area can be laborious to measure, taking more than 25 h per individual [7–9]. Here, we seek to develop new trait measures that encompass metabolic ecological function that can also be measured consistently for almost all sharks and rays [10].

Gill slits are external openings of the parabranchial cavity in sharks and rays (subclass Elasmobranchii) where exhalent oxygen-depleted water exits the body [11]. The structure of the gill slit arises from the extension of interbranchial septum, which supports the gill filaments and surface area where oxygen uptake occurs [11,12]. Thus, there is a direct functional morphological connection between the height of the external gill slit length and the corresponding internal gill surface area: shark gill slit height is related to gill surface areas such that species with larger gill surface areas have longer mean gill slit heights [9]. Specifically, gill slit height is positively correlated with (i) total gill surface area across species and (ii) parabranchial gill surface area (within an individual’s gill chambers or parabranchia) both within and across species [9]. Additionally, gill slit height measurements are generally equivalent whether measured on live specimens or anatomically accurate illustrations and can be made in a matter of minutes [9].

**Figure 1.**
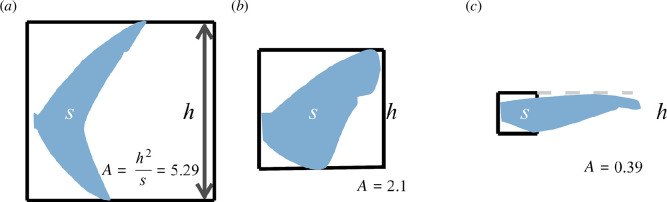
Schematic of how CFAR (*A*) is calculated (*h* ^2^/*s*). Black box represents the height of the caudal fin (*h*); the double-sided arrow in (*a*) squared (*h* ^2^) and blue tail shading are the surface areas (*s*). Panels show examples of the variation observed across shark species with (*a*) high (longfin mako *Isurus paucus*), (*b*) medium (prickly dogfish *Oxynotus bruniensis*) and (*c*) low (longfin catshark *Apristurus herklotsi*) levels of activity.

Similar to metabolic rate, gill morphology, including the gill slit height, is also correlated with activity, habitat (depth), and maximum size of a species. Collectively, we have termed these ‘ecological lifestyle’ traits [7,13,14]. The activity level of a species can be measured directly through swimming speed or indirectly through the caudal fin morphology [15–17]. The caudal fin aspect ratio (CFAR) is the ratio of *h*, the height of the caudal fin, and *s*, the area of the caudal fin, calculated as *A* = *h* ^2^/*s* (figure 1). CFAR is positively correlated with swimming speed in both teleosts and sharks [17,18]. Species with greater CFAR values are more active and have higher oxygen demands (i.e. metabolic rate [14,19]). Similarly, species that are pelagic with shallower average depths generally have faster swimming speeds, higher metabolic rates and larger gill surface area compared with deeper dwelling benthic species [7,14,20]. The maximum size of a species also has a predictable positive relationship with the metabolic rate (even beyond what can be accounted for by measurement of body mass, or the mass of the individual whose metabolic rate was measured), where larger-bodied species have higher metabolic rates [1,21].

Here, we examine whether gill slit height is broadly related to the ecological lifestyle of across sharks. Specifically, we ask four questions: (i) do more active sharks (those with greater CFAR) have proportionately longer gill slits, (ii) do larger-bodied sharks have proportionately longer gill slits, (iii) do deeper-dwelling sharks have proportionately shorter gill slits, and finally (iv) do all three ecological lifestyle traits together better explain variation in gill slit height than any individual component? Finally, we also consider whether endothermic species have longer gill slits. As metabolic rate is related to ecological lifestyle and gill surface area is strongly correlated with metabolic rate, then gill slit height should be similarly related to ecological lifestyle. We expect that more active, larger-bodied, shallow-water species will have greater gill slit height to accommodate greater oxygen demands (from higher metabolic rates). If true, rapid estimates of metabolic demand and by extension, life history traits, may be attainable without collecting live specimens, saving countless hours in laboratories and the costs associated with collecting the specimens.

## 2. Material and methods

### 2.1. Data collection

We measured total length (TL; snout to posterior of caudal fin), the height of each gill slit (following curvature), tail height (*h*; a vertical line from the top of the caudal fin to the bottom of the caudal fin) and tail area (*s*) from anatomically accurate illustrations from the first edition of *Sharks of the World* [22]. This field guide is the most complete in terms of biodiversity and its illustrations have been validated for morphological accuracy [7,9,18,22]. For each species of shark (*n* = 455), the lateral illustration was cropped and imported into ImageJ for measurement [23]. The height of each gill slit was measured and averaged across all gill slits (i.e. five, six, or seven gill slits) to generate a mean and divided by the TL of each species to calculate the gill slit height expressed as a proportion of total length, hereafter ‘GSH’. The mean gill slit height metric is suitable for comparing species with five gill slits but overlooks the extra gill area in the six-gill and seven-gill sharks. To represent the greater gill area of six- and seven-gill sharks, we also calculated the summed height of all gill slits. We also evaluated the degree to which 7 of the 8 regionally endothermic sharks had greater average gill slit heights and greater CFARs. We included the following seven lamniform sharks: common thresher shark (*Alopias vulpinus*) [24], smalltooth sandtiger (*Odontaspis ferox*) [25], both shortfin and longfin mako (*Isurus* spp.), porbeagle (*Lamna nasus*) and salmon shark (*Lamna ditropis*) as well as the white shark (*Carcharodon carcharias*) [26,27]. We omitted one regionally endothermic the basking shark (*Cetorhinus maximus*) [28], both frilled sharks (family Chlamydoselachidae) and the 20 angel shark species (family Squatinidae) because their gill slits curve under the body and hence were not completely visible in the illustrations. CFAR was calculated for each species defined as *A* = *h* ^2^/*s*, where *h* is the height of the caudal fin and *s* is the area of the caudal fin [7,29] (figure 1). Depth range (m) and maximum size (TL, cm) were collated for each species using a combination of published IUCN Red List assessments [30], species checklists [31] and *Sharks of the World* [22].

### 2.2. Statistical analysis

To answer our questions, we fitted four Bayesian linear models in a phylogenetic framework. Prior to analysis, GSH, CFAR, median depth and maximum size were all natural-log transformed and all variables were standardized to facilitate model convergence and comparison.

Each ecological lifestyle trait was modelled as a predictor of GSH separately:

,$$\ln\left(GSH\right)=\ln\left(\beta_{o}\right)+\beta_{i}\times \ln\left(x_{i}\right)$$

where GSH, the mean gill slit height of a species, is the response variable, *β*_o_ is the intercept, *x_i_* is a given ecological lifestyle trait and *β*_*i*_ is the slope of that ecological lifestyle trait. For Question 1 (do more active sharks have proportionately longer gill slits?), *x_i_* was CFAR, for Question 2 (do larger-bodied sharks have proportionately longer gill slits?), *x_i_* was maximum size, for Question 3 (do deeper-dwelling species have proportionately shorter gill slits?), *x_i_* was median depth and for Question 4 (do all three ecological lifestyle traits together better explain gill slit height?), the model included all three traits—CFAR, maximum size and median depth (the ‘global model’). To be sure that these related variables were not collinear in our model, we ensured the variance inflation factor (VIF) was less than five [32] (electronic supplementary material, table S2).

To account for the shared evolutionary history of traits between species, we included a random effect of phylogeny in all models that structured the residuals (i.e. error) by evolutionary distance between species [4,21]. To do so, we pruned a taxon-complete chondrichthyan phylogenetic tree (a molecular tree of 615 species of sharks, rays and chimaeras augmented with all remaining species based on taxonomic constraints; refer to Stein *et al.* [33] for more detail) to the species in our dataset (*n* = 455). To ensure our results were not biased owing to using the infilled taxon-complete tree versus the molecular tree, we also fitted all models with a random effect of the molecular tree derived from the 615-species molecular tree trimmed to shark species with molecular data only (*n* = 268).

The four models were fitted using the brms package in R v4.1.3 with four chains of 4000 iterations with 1000 warm-up iterations (5000 iterations total) [34,35]. In R code, this would look like GSH ~ CFAR + (1| gr(phylogeny, cov = A)), where gr() is a grouping function in the brms package and depending on the phylogeny nexus object and a covariance matrix A, which can be created using the ape package and the following code: A <- ape::vcv.phylo(phylogeny) [36]. Uninformative priors were used and convergence was assessed by ensuring R-hat values = 1 and effective sample size (ESS) >1000 [34]. All four models were compared to find the model with the most support and predictive ability using Pareto-smooth leave-one-out cross-validation [37]. Models with larger expected log pointwise predictive densities (elpd_loo) values (and lower looic values) have more support and higher predictive ability [37]. Generally, models with a difference of <2 looic units are considered to be equal in terms of fit and predictive ability and the most parsimonious model is usually preferred [37]. Using the molecular versus full phylogenetic tree did not affect the model parameters substantially; however, the lambda (λ) values were greater with the molecular tree (electronic supplementary material, tables S1 and S2).

## 3. Results

### 3.1. Do more-active sharks have larger gill slits?

**Figure 2.**
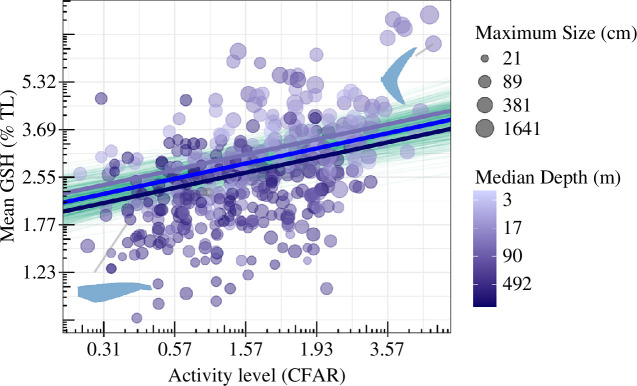
Relationship between activity level (CFAR) and mean gill slit height. Points are coloured by median depth and sized by maximum body size (total length, cm). Green lines are 500 random draws of conditional fit from the posterior distribution of the global model with blue lines indicating intercept differences at varying depths; blue is mean depth (90 m), dark blue and light blue are 1 s.d. in either direction (492 and 17 m). Inset silhouettes are of the longfin mako shark (*Isurus paucus*; top-right) and the longfin catshark (*Apristurus herklotsi*; bottom-left).

More-active sharks (those with greater CFARs) had larger mean gill slit heights (GSH; figure 2). CFAR ranged from 0.25 in the epaulette shark (*Hemiscyllium ocellatum*) to 5.29 in the endothermic longfin mako (*Isurus paucus*) and their respective GSH values were 2.62% TL and 7.14% TL. An average shark species had a CFAR value of 1.05 and was estimated to have a GSH of 3% TL (table 1). An increase in CFAR of 1 standard deviation (s.d., i.e. 0.89 CFAR units) had an estimated increase in GSH of 0.37 s.d. (i.e. an increase of 0.44% TL) (table 1).

**Table 1.** Model summary table comparing parameter estimates (with Bayesian credible intervals), posterior s.d., lambda, and *R* ^2^ based on the full phylogenetic tree (*n* = 455). The best model is highlighted in bold with model comparison details outlined in table 2.

	estimate (95% BCI)	posterior s.d.
model 1: GSH—activity; lambda (*λ*) = 0.63, Bayesian *R* ^2^ value (95% BCI) = 0.67 (0.61–0.71)		
intercept	0.03 (0.025–0.037)	0.27
slope—activity (CFAR)	0.37 (0.25–0.48)	0.06
model 2: GSH—maximum body size; lambda (*λ*) = 0.60, Bayesian *R* ^2^ value (95% BCI) = 0.65 (0.60–0.70)		
intercept	0.028 (0.024 to 0.034)	0.26
slope—maximum body size	0.32 (0.22 to 0.43)	0.05
model 3: GSH—median depth; lambda (*λ*) = 0.69, Bayesian *R* ^2^ value (95% BCI) = 0.66 (0.61–0.71)		
intercept	0.029 (0.024 to 0.037)	0.3
slope—median depth	−0.19 (−0.31 to −0.07)	0.06
**model 4: GSH—activity + maximum body size + median depth; lambda (*λ*) = 0.55, Bayesian *R* ^2^ value (95% BCI) = 0.66 (0.61–0.71**)		
**intercept**	**0.028 (0.024 to 0.034)**	**0.23**
**slope—activity (CFAR**)	**0.32 (0.21 to 0.43)**	**0.06**
**slope—maximum body size**	**0.23 (0.12 to 0.33)**	**0.05**
**slope—median depth**	**−0.19 (−0.3 to −0.08)**	**0.06**

### 3.2. Do larger-bodied sharks have larger gill slits?

GSH and maximum size were positively related such that species with larger maximum size had larger GSH. Maximum size ranged from the 15.7 cm TL Campeche catshark (*Parmaturus campechiensis*) to the 2000 cm TL whale shark (*Rhincodon typus*), with GSH values of 2.3% and 8.9% TL, respectively. An average shark species had a maximum size of 89 cm TL and an estimated GSH of 2.84% TL (table 1). GSH was estimated to increase by 0.33 s.d. units (0.35% TL) for an increase in 1 s.d. of maximum size of 95 cm TL (table 1).

### 3.3. Do deeper-dwelling sharks have smaller gill slits?

GSH and median depth were negatively related such that deeper-dwelling species had shorter average gill slit height (figure 2 and electronic supplementary material, figure S1). Median depth ranged from 2 m in the northern wobbegong (*Orectolobus wardi*) to 3325 m in the short-tail catshark (*Parmaturus bigus*), with GSH values of 3.0% and 1.4% TL, respectively. An average shark with a median depth of 90 m would have an estimated GSH of 2.94% TL (table 1). An increase in depth by 1 s.d. (402 m deeper) would result in a reduction in GSH of 0.19 s.d. (i.e. decrease by 0.2% TL; table 1).

### 3.4. Does ecological lifestyle explain GSH better than its components separately?

**Figure 3.**
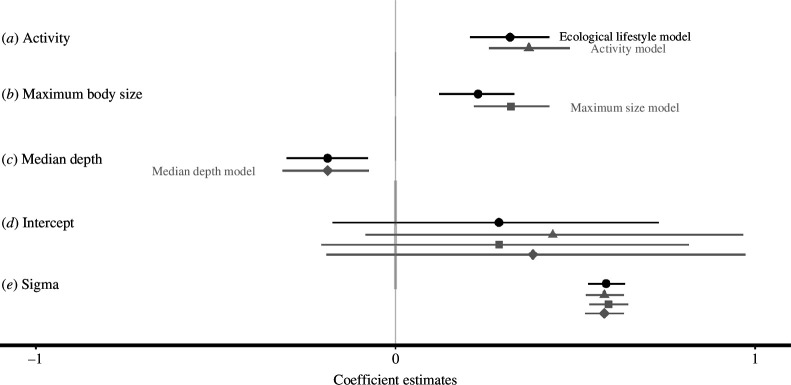
Model coefficient estimates for each of the four models evaluated. Ecological lifestyle (global) model estimates are in black (circles) while univariate model estimates are in grey (activity—triangles, maximum body size—squares and median depth—diamonds). Lines for each coefficient indicate the 95% credible interval.

The effect of CFAR on GSH was positive and greater (*β* = 0.31) than the effect of maximum size (*β* = 0.23), whereas median depth had a negative effect on GSH (*β* = −0.19; figure 2, table 1). The global model had the most support out of the four models (*elpd_loo* = −459.17; table 2). The models with individual ecological lifestyle traits have less support than the global model, with the model with just CFAR being the second-best model (table 2). Nevertheless, the individual trait models estimated similar parameter estimates (grey coefficients; figure 3) to the global model (black coefficients; figure 3).

**Table 2.** Model comparison table with the gill slit height models for the full phylogenetic tree (*n* = 455). The model with the best fit and support is highlighted in bold text.

model	*p*_loo_	looic	elpd_loo_	se_elpd_loo_	elpd_diff_	weight
model 1: activity (CFAR)	18.23	935.2	−467.6	18.23	−8.44	0.10
model 2: maximum body size	104.93	944.47	−472.23	18.1	−13.07	0.00
model 3: median depth	125.93	957.88	−478.94	19.43	−18.31	0.21
**model 4: global model**	**94.62**	**918.33**	**−459.17**	**17.96**	**0**	**0.55**
model 5: global model + endothermy	93.45	924.5	−462.25	18.79	−3.08	0.13

## 4. Discussion

Overall, we found that gill slit height was closely related to ecological lifestyle. Specifically, sharks with greater mean gill slit heights were more active (and had greater CFARs, larger maximum sizes and shallower depth ranges; figure 2). Furthermore, we found that the global model that considered all three ecological lifestyle traits together best-explained gill slit height compared with any model with a given ecological lifestyle trait on its own (table 2). Next, we consider four points: (i) the connection between activity and oxygen demand, (ii) the connection between maximum size and gill slit height, (iii) the evolutionary response to reduced oxygen in the deep sea, and finally (iv) future directions.

### 4.1. Activity and oxygen demand

Activity level has long been known to be positively correlated with metabolic rate and gill surface area across mainly teleost fishes [7,13,38]. Here, we show that activity level is also positively related to gill slit height across shark species (figure 2), suggesting that measures of gill slit height, along with CFAR, could be used to infer metabolic rates, activity levels, and typical swimming speeds [7,18]. Previous work has shown that endothermic fishes cruise around 1.6 times faster than ectotherms, accounting for the effect of body mass on temperature and speed [15]. Here, we show that gill slit height is also greater (0.47 s.d. units and hence by 0.5% of TL) in endothermic sharks compared with the others while accounting for activity (CFAR), maximum body size, and median depth (electronic supplementary material, table S5). One hypothesis that may explain these positive relationships is that species that are more active, and/or can support endothermy, have a higher oxygen demand (and larger gill slits and presumably greater gill surface area) to support higher metabolic demand [6,14,39].

### 4.2. Maximum body size and gill slit height

Larger-bodied species have larger gill slit heights and this relationship hints at a more general one between gill morphology and body size across sharks. Bigman *et al*. [7] found that larger-bodied sharks (based on maximum size) had larger gill surface areas, which supports the idea that gill slit height would also be larger for larger-bodied species. GSH in this study is scaled to be proportional to total length, so sharks with greater GSH have larger GSH in relation to their body length. In this sense, if each species had the same total length, variation in GSH may correspond to differences in maximum or asymptotic sizes, with larger-bodied species having larger GSH and smaller-bodied species having smaller GSH. This may indicate that species with larger GSH are expending extra resources on maintaining larger gills to potentially reach larger asymptotic sizes (i.e. facilitate growth). This idea is consistent with the gill oxygen limitation theory, which argues that growth and other processes requiring energy from aerobic metabolism are limited by oxygen consumption (diffusion) at the gills because the scaling of gill surface area with body mass is geometrically constrained to be less than one [40,41]. However, recent work has found mixed support for the strong causality of this theory [42–46]. For example, Lefevre *et al*. [43] argue that the folded nature of gill surface area does not follow strict geometric constraints (i.e. they can scale with body mass ontogenetically to ≥1). Marshall & White [45] reiterate that decades of work on life history theory have revealed that growth, size, and metabolic level are together shaped by selection, and Bigman *et al*. [39] show that gill surface area does not explain as much variation in growth and size across fishes (including elasmobranchs) as activity level indexed by CFAR. Despite the current debate regarding the degree to which gill morphology has a causal effect on aerobic metabolism, the (mean) ontogenetic scaling of gill surface area does seem to be less than one and very similar to the scaling of metabolic rate resulting in a useful correlation [7,38,47]. In sharks, for example, ontogenetic scaling of gill surface area scales with body mass according to a lower law relationship (i.e. GSA ~ *M* ^*b*^, where the exponent *b* averages around 0.85 (range 0.75–0.9) [7,39,42]). Furthermore, species that grow to larger maximum sizes maintain larger-than-average gills throughout their lifetime, which may allow for the necessary oxygen uptake for growth, maintenance and reproduction [7].

### 4.3. The evolutionary response to reduced oxygen in the deep sea

Deepwater sharks could provide some insight into the connection between metabolic rate and functional morphology. Deepwater species tend to be smaller-bodied with smaller gill slit heights and low activity levels (lower CFAR; figure 2 darker shades of blue). There are multiple abiotic challenges to living in the deep sea (>200 m), most notably the low temperature and oxygen concentration. Species that live in deeper waters are often in environments with much lower temperatures and oxygen levels than pelagic and coastal surface waters [48–50].

The evolutionary response to reduced oxygen in the deep sea could be (i) increased gill area, (ii) reduced body size (hence growth rates and reproduction) or (iii) reduced activity, or some combination of the three responses. Generally, in contrast to the first idea above, deepwater sharks have shorter gill slit heights. However, in some species, gill slit height is elongated by extending the gill slits under the body to end only at the midline, as seen in frilled sharks (family Chlamydoselachidae) and the filter-feeding basking shark. A small number of deepwater shark species have increased the number of parabranchii and gill slits, as seen in the cow sharks (Hexanchidae), such as the bluntnose sixgill shark (*Hexanchus griseus*), and broadnose sevengill shark (*Notorhynchus cepedianus*), as well as the sixgill stingray (*Hexatrygon bickelli*). While these species have additional gill slits, using their mean gill slit height compared with summed gill slit height had little effect on our overall results (electronic supplementary material, figures S3 and S4; table S4). Another way to increase gill area is to increase head size laterally to accommodate larger gill surface area as is exemplified in the lollipop shark (*Cephalurus cephalurus*) and filetail catshark (*Parmaturus xaniurus*), which inhabits anoxic water (with oxygen concentrations of <1% of that found in surface waters; 1.6 μmol kg^−1^), and as such, is a low-oxygen specialist [26]. Similarly, the bigeye thresher (*Alopias superciliosus*) is the only thresher that forages in the deep Oxygen Minimum Zone and has the largest known gill surface area of any elasmobranch with an expanded branchial cavity to accommodate the larger gill surface area [51]. So while some species have an extra gill arch or two or enlarged heads to cope with anoxia, overall, the general pattern is of smaller, rather than larger gill slit heights (and presumably gill surface area), in deeper sharks.

Second, additional adaptations to life in low-oxygen environments include the inevitable thermodynamic reduction in metabolic rate and activity in response to lower temperatures. The reduced metabolic rate of deepwater species is thought to underlie reduced growth rates, reproductive output and population growth rates compared with shallow-water species [21,48,49,52]. Deepwater sharks tend to be small, slower growing, have lower reproductive output, and generally have slower life histories [49,53,54]. Deepwater habitats exhibit some of the smallest shark species, suggesting that over evolutionary time, deepwater species have reduced their maximum size, not only as a result of the thermodynamic response to lower temperatures but also we hypothesize to ensure they are not in a constant state of hypoxia in deeper water. Third, deepwater sharks tend to have much lower activity levels and swimming speeds [55]. Indeed, the most active deepwater sharks, the lantern sharks (Etmopteridae), are also among the smallest species of sharks in the world [55], hence we hypothesize that deepwater sharks can be small and active or large and inactive but not both.

Another possible limiting factor in the deep is prey availability and encounter rates (visual-interactions hypothesis [52]). With reduced (or no) visible light, the chances of encountering prey are much lower than in shallow, light-filled waters and, thus, reduced resources for metabolism. The reduced resources at depth and energetic costs of a lipid-rich and buoyant liver in chondrichthyans may explain why they are largely absent below 3000 m [56]. Together, the low temperatures, low oxygen and low food availability could explain the smaller gills and slower pace of life observed in deep-sea sharks as well as innovations such as the evolution of more gill slits and larger heads [49]. Clearly, understanding the evolutionary trajectory of these adaptations is a key question, and according to the Compagno [57] hypothesis, deepwater sharks are ancestral, and hence the hypothesis to test is whether the low activity, smaller gill lifestyle are ancestral, and the general evolutionary trajectory has been toward more active larger gill lifestyles of coastal and pelagic species.

### 4.4. Future directions

Although gill slit heights measured from anatomically accurate illustrations in field guides are comparable to those measured on physical specimens, we recognize that our morphological measurements (GSH and CFAR) from such illustrations may not be identical to those from physical specimens. GSH can vary within species in the range of ~1.3% fork length (i.e. length from snout to fork of caudal fin), though we have confirmed the relationship between gill slit height of field guide drawings closely matches laboratory measurements in five species of carcharhinid [9]. Field guides are routinely used as a source of data in scholarly publications, and specifically, ecological studies [58]. Such approaches offer the advantage of requiring relatively low effort and cost and can be applied consistently and repeatedly across the whole lineage. There are three alternative validation approaches that are expensive and laborious, but nevertheless complementary and would build trust in these simple trait measures. First, by opportunistically measuring live specimens or individuals that have been recently captured from ongoing fieldwork, possibly sourced through a ‘citizen’ science project. Second, museum specimens can be used to validate the accuracy of field guide illustrations, but they have been preserved for many years and may have experienced shrinkage from fixing agents as well as damage from multiple specimens in cramped storage containers. Third, there is increasing development of underwater laser photogrammetry measuring techniques, to estimate maximum size using paired lasers to provide a reference size within the field of view of a video camera. These data could also be used to measure gill slit height and CFAR [59,60].

## 5. Conclusion

We have developed simple, easily measured, cost-effective trait relationships linking metabolic morphology and ecological lifestyle across sharks. These measures can help shed light on the metabolic ecology of a large lineage of marine fauna that have very few and hard-to-gather metabolic and life history data [8]. Our previous work has shown that metabolic rates are weakly related to life history traits, such as growth rate and age at maturation; however, the exploration of these relationships is constrained by the limited availability of metabolic rate data [21,53]. We hope that these metabolic morphology traits will improve predictions of life history traits and maximum intrinsic population growth rate while minimizing the collection of live specimens or spending countless hours in laboratories. Many species are data-poor, particularly on the population scale at which management occurs [61]. The development of metabolic morphology traits will go a long way to expand the breadth of species that can be evaluated using ecological risk assessments including species that are rarely observed or have only been observed a small number of times.

## Data Availability

Data and code are available online at [62].
